# Clustering Patterns of Unhealthy Lifestyle Behaviours Among Adolescents: A Multilevel Analysis of a Nationally Representative School-Based Survey from 73 Countries

**DOI:** 10.3390/nu17040609

**Published:** 2025-02-07

**Authors:** Yohannes Tekalegn Efa, David Roder, Zumin Shi, Ming Li

**Affiliations:** 1 Cancer Epidemiology and Population Health Research Group, Allied Health and Human Performance, University of South Australia, Adelaide 5000, Australia; david.roder@unisa.edu.au (D.R.); or m.li3@uq.edu.au (M.L.); 2 South Australian Health and Medical Research Institute (SAHMRI), Adelaide 5000, Australia; 3 Department of Nutrition Sciences, College of Health Sciences, QU Health, Qatar University, Doha P.O. Box 2713, Qatar; zumin@qu.edu.qa; 4 Faculty of Medicine, University of Queensland, Brisbane 4006, Australia

**Keywords:** lifestyle, dietary habit, physical activity, sedentary behaviour, clustering pattern, adolescents, global school-based student health survey

## Abstract

**Background**: Adolescence is a crucial stage when young people adopt various lifestyle behaviours that can impact their health. However, the broader determinants of these behaviours remain underexplored. This study aimed to investigate clustering patterns of lifestyle behaviours, including physical activity, sedentary behaviour, and dietary habits, among adolescents and examine their determinants across individual, community, and societal levels. **Methods**: This study utilised nationally representative Global School-based Student Health Survey data. The lifestyle behaviours were dichotomised based on specific definitions, and the clustering patterns of multiple unhealthy behaviours were compared at various levels of factors. Multilevel logistic regression analysis was employed to identify individual, community, and societal level determinants of multiple unhealthy behaviours. **Results**: The study included 293,770 adolescents from 73 countries and territories across five World Health Organization (WHO) regions. The overall prevalence of one, two, three, four, and five unhealthy behaviours was 6.9%, 29.9%, 36.5%, 21.5%, and 4.5%, respectively. The odds of multiple unhealthy behaviours (defined by ≥4) increase with age and are higher among female adolescents [(AOR: 1.06, 95% CI:1.05, 1.07); (AOR: 1.16, 95% CI: 1.14, 1.19)], respectively. Adolescents from supportive families, peer environments, or food-secure households had lower odds of engaging in these behaviours [(AOR: 0.84, 95% CI: 0.82, 0.86); (AOR: 0.96, 95% CI: 0.94, 0.98); (AOR: 0.91, 95% CI: 0.87, 0.95)], respectively. The odds of exhibiting multiple unhealthy behaviours were significantly higher among adolescents in countries with high (0.7–0.79) and very high (0.8–1.0) Human Development Indexes (HDI) compared to those in low (<0.55) HDI countries [(AOR: 1.84, 95% CI: 1.16, 2.94); (AOR: 3.00, 95% CI: 1.48, 6.08)], respectively. **Conclusions**: The study findings reveal that multiple unhealthy lifestyle behaviours, including insufficient physical activity, sedentary behaviour, inadequate fruit and vegetable consumption, frequent soft drink intake, and fast-food consumption, are globally prevalent among adolescents. These behaviours have distinct clustering patterns associated with individual characteristics, family and peer environments, and broader socio-economic and societal contexts.

## 1. Introduction

Adolescence is a transition phase of life from childhood to adulthood, marked by rapid physical, cognitive, and socio-emotional transformations essential for establishing a solid foundation for optimal health [1,2,3]. Lifestyle behaviours are daily activities shaped by an individual’s values, knowledge, and norms within a broader cultural and socio-economic context [4]. Adopting a healthy lifestyle early in life is strongly associated with a longer life expectancy free from chronic non-communicable diseases (NCDs) such as diabetes, cardiovascular disease, and certain cancers [5,6]. NCDs were previously considered as diseases of adulthood. Nowadays, it is affecting the younger population due to changes in lifestyle habits in adolescents. The World Health Organization (WHO) has identified unhealthy lifestyle habits during adolescence, such as insufficient physical activity, unhealthy diets, and harmful use of alcohol and tobacco, as key contributors to the rising burden of NCDs [7]. The findings from the global burden of disease highlight that NCDs accounted for 86.4% of all years lived with disability (YLDs) and 38.8% of total deaths in adolescents aged 10–24 years in 2019 [8].

Unhealthy lifestyle behaviours, such as physical inactivity, insufficient consumption of fruits and vegetables, sedentary behaviour, fast-food consumption, and soft drink intake, are prevalent among adolescents [9,10,11]. Globally, 86% of adolescents worldwide do not consume the recommended amount of fruits and vegetables, 55% consume fast-foods at least once a week [12], 54% consume carbonated soft drinks daily [13], 85% do not achieve WHO’s guideline of at least 60 min of physical activity per day, and 30% report engaging in sedentary behaviour [11].

About 82% of adolescents aged 11–17 years report engaging in two or more unhealthy lifestyle behaviours, including low fruit and vegetable intake, physical inactivity, high sedentary behaviour, overweight or obesity, alcohol intake, or smoking [11]. The co-occurrence or clustering of multiple unhealthy lifestyle behaviours increases the risk for NCDs compared to individual behaviours alone [14,15,16,17].

Studies have shown that unhealthy lifestyle clusters significantly increase with age and are more prevalent among female adolescents [9,18,19]. Social support systems, including family and peers, are crucial in shaping adolescent health behaviours. These support networks can provide emotional and practical resources reinforcing healthier choices [20,21,22,23]. Additionally, differences in country-level contexts such as geographical regions and development indexes, cultural norms, and public health policies across countries profoundly impact the adaptation of lifestyle behaviours among adolescents [24,25,26,27,28].

Most previous research has focused primarily on individual level factors or has been limited to a single country or region, missing the broader context of these behaviours [18,29,30,31]. Research investigating the interaction of multilevel factors, including individual characteristics and socio-economic development across countries, and their influence on the clustering of unhealthy behaviours among adolescents is still scarce. To address these gaps, this study aimed to 1. assess the clustering of patterns of five unhealthy lifestyle behaviours, including insufficient physical activity, sedentary behaviour, inadequate consumption of fruit and vegetables, frequent consumption of carbonated soft drinks, and frequent fast-food consumption; and 2. investigate multilevel factors that are associated with the clustering of unhealthy lifestyle behaviours. These factors included individual sociodemographic characteristics, family, peer support, and country-level factors such as geographic regions and socio-economic development. The study hypothesised that:

**H1.**  *Unhealthy behaviours clustered with high prevalence among school-aged adolescents.*

**H2.**  *The clustering of unhealthy lifestyle behaviours varies by age and sex, with stronger family and peer support linked to lower odds of unhealthy behaviour clusters.*

**H3.**  *Country-level socio-economic development is associated with the clustering patterns*.

Addressing this gap is essential for informing public health policies and targeted interventions to reduce these risks in adolescent populations globally.

## 2. Methods

### 2.1. Data Source and Study Population

This study utilised publicly accessible data from the Global School-based Student Health Survey (GSHS), an ongoing international collaborative surveillance initiative jointly conducted by the World Health Organization (WHO) and the U.S. Centers for Disease Control and Prevention (CDC). The survey covers various countries and territories across different socio-economic and geographical regions worldwide. The WHO provides technical assistance to countries throughout the planning and implementation phases, including survey questionnaire development, sample design, training of survey coordinators, data editing and weighting, and facilitating funding and other resources. Two types of training were provided to survey coordinators in each participating country. The first training focuses on building capacity for sampling and survey administration procedures to generate high-quality data. The second training occurs after fieldwork and centres on data analysis to produce country-specific reports and fact sheets [32].

A two-stage cluster sampling design was used to select a representative sample of school-aged children and adolescents within each country. In the first stage, schools were selected with a probability proportional to their enrolment size; in the second stage, classes were randomly selected, and all students enrolled in those classes were eligible to participate in the study.

The GSHS employs a self-administered questionnaire designed to be completed within a single class period, with students recording their responses on a computer-scannable survey tool. Data from all participating countries are centrally scanned and processed at the CDC, using uniform data editing procedures to ensure consistency and comparability across datasets. Information collected from the questionnaire includes sociodemographic characteristics such as age, sex, household food security, and family and peer support. In addition, the questionnaire collects 10 main sections that cover various behavioural aspects, including physical activity, dietary habits, tobacco use, alcohol and other drug use, and mental health. According to the guidelines, each participating country must select at least six of these ten main modules for inclusion in the national survey. Additionally, each country translates the survey tools into its local language(s). Further details on the survey methodology and questionnaire can be found on the WHO website [33].

**Figure 1:**
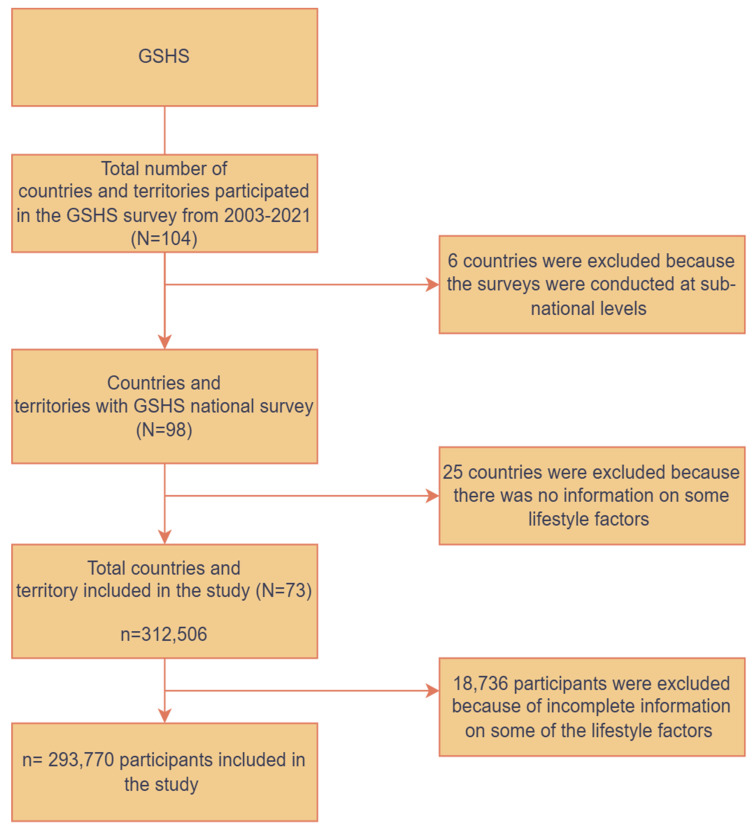
Flow diagram illustrating sample selection process.

About 104 countries and territories implemented the GSHS between 2003 and 2021. We selected only the most recent data for countries with multiple survey rounds. Surveys from six countries (China, Colombia, Ecuador, Venezuela, Zimbabwe, and the Occupied Palestinian Territory) were excluded from the study because they were conducted at sub-national levels and may not have been nationally representative. Among the remaining 98 countries with nationally representative surveys, we excluded an additional 25 because they did not measure all 5 lifestyle behaviours used as the primary outcome variables in this study: physical activity, leisure time sedentary behaviour, fruit and vegetable consumption, carbonated soft drink consumption, and fast-food consumption. Furthermore, 18,736 cases with missing data on the 5 lifestyle behaviours were excluded. Finally, we pooled the nationally representative surveys from 73 countries, resulting in a sample of 293,770 adolescents (Figure 1).

### 2.2. Study Outcome

#### Lifestyle Behaviours

This study included five lifestyle behaviours commonly measured by most participating countries: physical activity, leisure-time sedentary behaviour, fruit and vegetable consumption, carbonated soft drink consumption, and fast-food consumption. The behaviours, corresponding self-reported questions, and definitions are summarised below (Table 1).

### 2.3. Study Covariates

#### 2.3.1. Individual- and Household-Level Covariates

Age, sex, household food insecurity, family or peer support, and weight status were included as covariates in the study. Food insecurity was a proxy for household socio-economic status since it was not directly measured in the GSHS. It was assessed using the question, “During the past 30 days, how often did you go hungry because there was not enough food in your home?”, with response options of “never”, “rarely”, “sometimes”, “most of the time”, and “always”. Those who responded with “most of the time” or “always” were classified as experiencing food insecurity [39]. Peer support was measured with the question, “During the past 30 days, how often were most of the students in your school kind and helpful?”. Family support was evaluated using the question, “During the past 30 days, how often did your parents or guardians appear to understand your problems and worries?”. For both questions, the response options were “never”, “rarely”, “sometimes”, “most of the time”, and “always”. Responses were categorised as “low level” of peer or family support for those who answered “sometimes”, “rarely”, or “never”, and as “high level” for those who responded “always” or “most of the time” [40]. Trained survey staff measured weight and height. Age- and sex-specific BMI values were calculated, and participants were classified as overweight/obese or not using the WHO growth reference [41].

#### 2.3.2. Country-Level Socio-Economic Covariates

Country-level identifiers such as WHO regional classifications and national socio-economic and development indexes such as World Bank income classification (https://data.worldbank.org, accessed on 30 April 2024) and Human Development Index (HDI) from the United Nations Development Programme (UNDP) data centre (https://hdr.undp.org/data-center, accessed on 24 April 2024) were matched and linked with a survey dataset at the country-level. The HDI values reflect achievements in living standards, health, and education, ranging from 0 to 1, with higher values indicating higher development. Countries are classified as follows: very high (0.80–1.0), high (0.70–0.79), medium (0.55–0.69), and low (<0.55) human development [42].

### 2.4. Statistical Analysis

#### 2.4.1. Data Preparation and Descriptive Analyses of Clusters of Lifestyle Behaviours

Data were cleaned and processed using the statistical software R version 4.4.0 [43] and STATA/SE version 18 [44]. Before statistical analyses, the GSHS weighting factors were applied to adjust for variations in the probability of selection and response rates. The survey design was specified in STATA and incorporated sampling weights, strata, and primary sampling units (PSUs). Each of the five lifestyle behaviours was dichotomised according to specific definitions, with a code of “1” indicating the presence of an unhealthy behaviour and “0” indicating its absence. The clustering patterns of these unhealthy lifestyle behaviours were descriptively summarised using an “upsetplot” function in STATA. The “upsetplot” function was used to create visual representations of the intersections between different sets of behaviours, allowing for the exploration of clustering of multiple unhealthy behaviours within an individual [45]. The weighted prevalence of each of the five unhealthy behaviours was calculated. Additionally, the total number of unhealthy behaviours was summed across the five examined behaviours, resulting in total values ranging from “0” to “5”. A value of “0” indicates the absence of all five unhealthy behaviours. In contrast, a value of “5” means the presence of all five.

#### 2.4.2. Multilevel Logistic Regression Analyses

The co-occurring unhealthy lifestyle behaviours were categorised into two categories: engaging in four or more unhealthy lifestyle behaviours versus fewer for subsequent logistic regression analysis with multilevel logistic regression to accommodate the hierarchical data structure, where students are nested within schools, and schools are nested within countries [46]. Three models incorporating individual- and country-level variables were fitted. Model I is the empty model without any covariates. Model I is the intercept-only model that measures the variation in the odds of the outcome across countries, serving as a baseline for comparison with more complex models. Model II includes individual- and household-level covariates. Model III includes covariates in model II and country-level socio-economic identifiers. Model III is the final model reported and used to estimate the measures of association.

The fixed effects were reported as adjusted odds ratio (AOR) and 95% confidence intervals (CIs). Multicollinearity was checked by variance inflation factors (VIFs). Statistical significance was declared at *p* < 0.05.

Random effects (measures of variation) at individual and country levels were presented in terms of intra-class correlation (ICC), percentage change in variance (PCV), and median odds ratio (MOR) [47,48]. The ICC explains how much of the variance in unhealthy lifestyle behaviours is attributable to differences between countries. A higher ICC indicates that a significant proportion of the variability is due to differences between countries. In comparison, a lower ICC implies that most of the variability is at the individual level, with less influence from country-level variables. The PCV measures the percentage reduction in country-level variance when adding predictors to the model. It shows how much of the between-group (country-level) variability is explained by adding the individual-, household-, or country-level predictors. It is a valuable measure to assess the model’s effectiveness in reducing unexplained variance at the group-level. When randomly selecting two areas, MOR represents the median value of the odds ratio between the highest-risk and lowest-risk areas. It reflects the typical increase in risk that a person would experience if they moved from a lower-risk area to a higher-risk area [47].

Model fit was evaluated using Akaike information criterion (AIC), deviance, and log-likelihood (LL) statistics. Among the three fitted models, the model with the highest log-likelihood and the lowest AIC and deviance was considered the best-fitting model [49].

## 3. Results

### 3.1. Description of Surveys Included in Study

**Figure 2:**
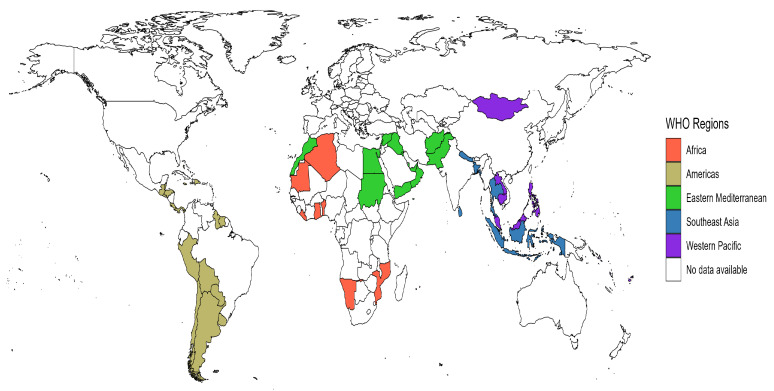
The 73 countries included in the study by the WHO region.

The surveys included data from 73 countries, with 293,770 adolescents across 5 WHO regions (Figure 2). The Americas region is represented by 26 countries with 110,699 participants, followed by the Western Pacific with 71,782 participants from 17 countries and the Eastern Mediterranean with 50,623 participants from 14 countries. Moreover, 10 countries with 29,163 participants represent Africa, and the Southeast Asia region has 31,503 participants from 6 countries. Most countries achieved survey response rates of above 70% (Table S1).

### 3.2. The Characteristics of the Study Participants

The study participants ranged from 12 to 17 years, with 51.1% male and 52% under 15. Moreover, 57.3% and 63.8% of participants reported low peer and family support levels, respectively. Additionally, 96.7% of the participants were from low- and middle-income countries. Notable variations were observed across the WHO regions regarding age, weight status, peer and family support, household food security, country-level income, and the Human Development Index (HDI) (Table 2).

### 3.3. Prevalence of Unhealthy Lifestyle Behaviours by Region

**Figure 3:**
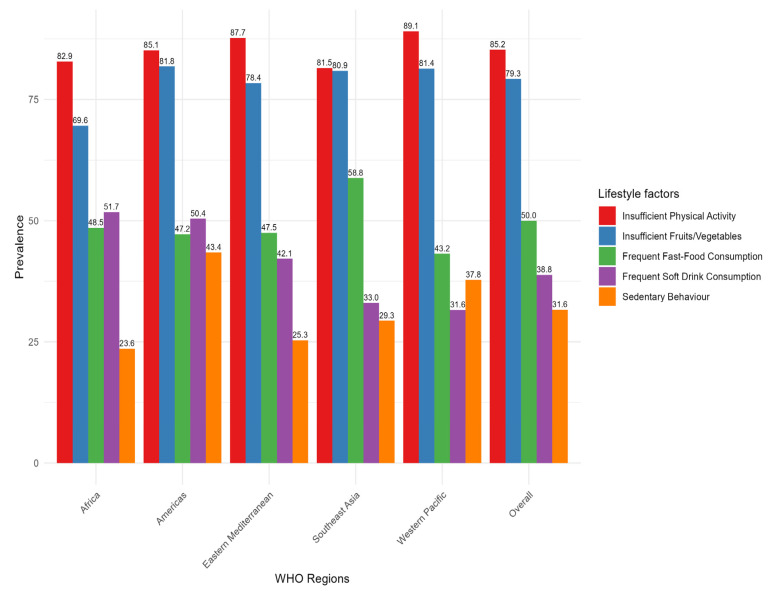
Prevalence of individual unhealthy lifestyle behaviours by WHO regions among adolescents aged 12–17 across 73 countries.

The overall prevalence of insufficient physical activity was 85.2%, ranging from 82.9% in Africa to 89.1% in the Western Pacific. Inadequate fruit and vegetable consumption was the second most common, with an overall prevalence of 79.3%. The observed prevalence ranges from 69.5% in Africa to 81.8% in the Americas. The prevalence of fast-food consumption was 50%, with the highest in Southeast Asia (58.8%) and the lowest in the Western Pacific (43.2%). The overall prevalence of frequent soft drink consumption was 38.8%, with the highest observed in Africa (51.8%) and the Americas (50.4%), compared to the lowest in the Western Pacific (31.6%). Among the unhealthy lifestyle behaviours studied, the prevalence of sedentary behaviour was the lowest, with an overall prevalence of 31.6%. The prevalence ranges from 23.6% in Africa to 43.4% in the Americas (Figure 3, Table S2).

**Figure 4:**
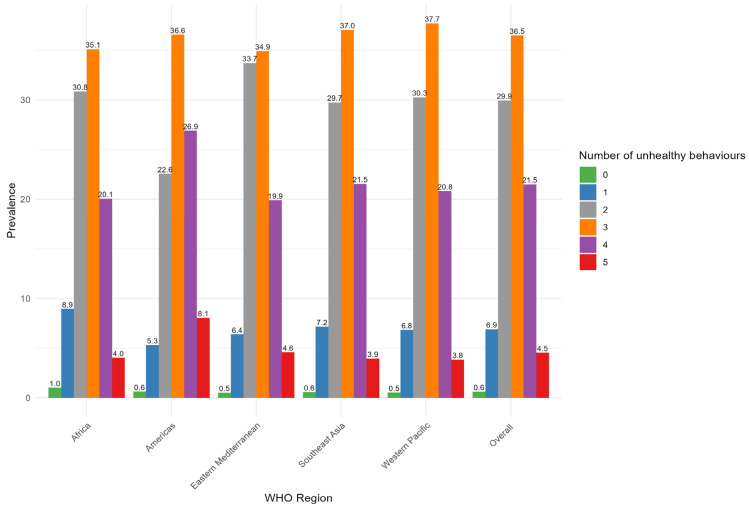
Prevalence of one more unhealthy behaviour by WHO region among adolescents aged 12–17 across 73 countries.

The proportion of individuals not having unhealthy behaviours is consistently less than 1% across all regions, with an overall prevalence of 0.6%. The overall prevalence of one, two, three, four, and five unhealthy behaviours were 6.9%, 29.9%, 36.5%, 21.5%, and 4.5%, respectively (Figure 4, Table S3).

### 3.4. Clustering Patterns of Unhealthy Lifestyle Behaviours

**Figure 5:**
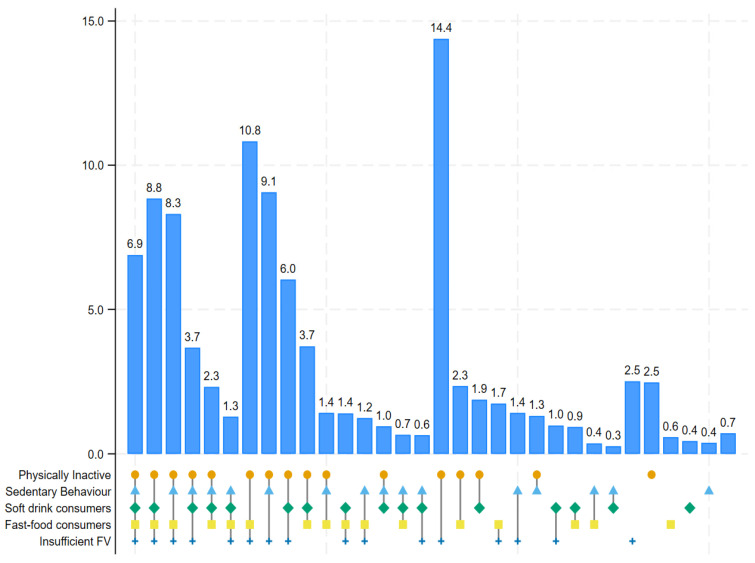
Clustering of unhealthy lifestyle behaviours among adolescents aged 12–17 years across 73 countries (unweighted prevalences reported using upsetplot function in Stata).

A total of 32 unique clustering patterns were identified. The simultaneous presence of all five behaviours was observed in 6.9% of the participants. Five different combinations of four concurrent unhealthy behaviours were identified, with prevalences ranging from 1.3% to 8.8%. For the co-occurrence of 3 unhealthy behaviours, 10 unique combinations were identified, with prevalences ranging from 0.6% to 10.8%. The prevalence of any two concurrent unhealthy behaviours varied widely, ranging from 0.3% to 14.4% (Figure 5).

**Figure 6:**
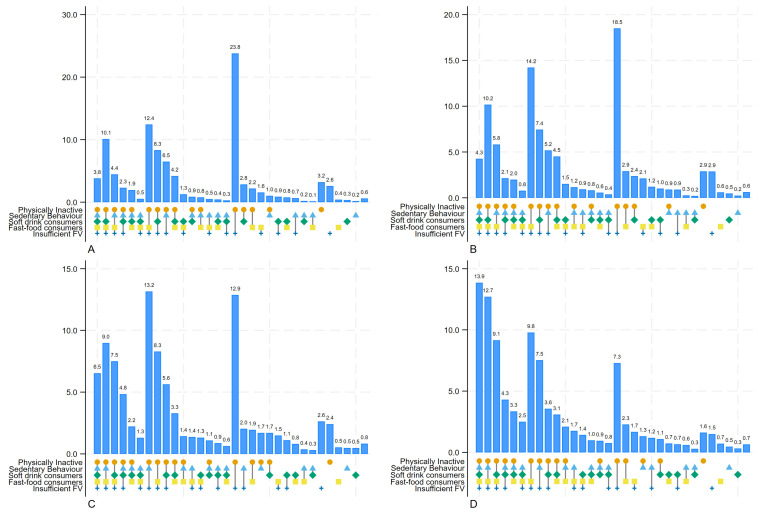
Clustering of unhealthy lifestyle behaviours by World Bank Income among adolescents aged 12–17 across 73 countries (unweighted prevalences reported using upsetplot function in Stata). (**A**) Low-income countries, (**B**) Lower middle-income countries, (**C**) Upper middle-income countries, (**D**) High-income countries.

The prevalence of five concurrent unhealthy behaviours was 3.8% in low-income countries, rising to 4.3%, 6.5%, and 13.9% in lower-middle, upper middle, and high-income countries, respectively. Conversely, the prevalence of concurrent insufficient physical activity and inadequate fruit and/or vegetable consumption was 23.8% in low-income countries, decreasing to 18.5%, 12.9%, and 7.3% in lower-middle, upper middle, and high-income countries (Figure 6). The patterns of unhealthy behaviours are consistent across both sexes, with females exhibiting slightly higher prevalences (Table S4).

The overall prevalence of adolescents exhibiting four or more unhealthy behaviours is 26.1%. Females show a slightly higher prevalence than males. The prevalence significantly varies by various factors at the individual, household, relationship, and country levels (Table 3).

### 3.5. Multilevel Factors Associated with Clustering of Four or More Unhealthy Behaviours

#### 3.5.1. Random Effects Results

The baseline model without predictor variables shows significant country-level variation in the prevalence of exhibiting four or more unhealthy behaviours, with a group-level (country-level) variance of τ^2^ = 0.38 (*p* < 0.0001). About 10.2% of the variability can be attributed to differences between countries (ICC = 0.102). The between-country variability declined in the subsequent models from 10.2% in model I, 9.8% in model II, and 3.4% in model III. The proportional change in variance (PCV) indicates that about 68.2% of between-country variability could be explained by adding individual-, household-, and country-level covariates in model III. The median odds ratio (MOR) for exhibiting four or more unhealthy lifestyle behaviours was 2.04 (95% CI: 1.83, 2.31) in model I, indicating that, on average, a person moving from a low-risk country to a high-risk country would experience 2.04 times higher odds of having four or more unhealthy behaviours. After adjusting for individual-, household-, or country-level predictors, MOR significantly reduced to 1.49 (95% CI: 1.39, 1.61) in model III. The model fit statistics such as Akaike information criterion (AIC), deviance, and log-likelihood suggest significant improvement in successive models, indicating model III is the best-fitting model. Therefore, model III is selected to report the individual-, household-, and country-level factors associated with the outcome variable (Table 4).

#### 3.5.2. Fixed Effects Results

The multilevel logistic regression analysis identified individual-, household-, and country-level factors. Age, sex, peer support, family support, household food security, WHO region, and Human Development Index (HDI) were significantly associated with exhibiting four or more unhealthy behaviours.

For every one-year increase in age, the odds of exhibiting four or more unhealthy lifestyle behaviours increase by 6% (AOR = 1.06, 95% CI: 1.05, 1.07). Female participants had 16% higher odds of exhibiting the outcome compared to males (AOR = 1.16, 95% CI: 1.14, 1.19).

Adolescents with supportive peers in their school had 4% decreased odds of exhibiting four or more unhealthy lifestyle behaviours than their counterparts (AOR = 0.96, 95% CI: 0.94, 0.98). The odds of exhibiting four or more unhealthy lifestyle behaviours decreased by 16% among adolescents with high family support compared to adolescents with low family support (AOR = 0.84, 95% CI: 0.82, 0.86). Adolescents from food-secure households had 9% lower odds of engaging in four or more unhealthy behaviours than those from food-insecure households (AOR= 0.91, 95% CI: 0.87, 0.95).

Compared to adolescents in the Western Pacific region, those in the African region, Americas, and Eastern Mediterranean region had significantly higher odds of exhibiting four or more unhealthy lifestyle behaviours, with adjusted odds ratios (AOR) and 95% CI of 1.49 (1.04, 2.15), 1.78 (1.37, 2.30), and 1.42 (1.06, 1.90), respectively. In contrast, Southeast Asian adolescents did not show a statistically significant difference (AOR = 1.14, 95% CI: 0.78, 1.65).

Compared to countries with a low Human Development Index (HDI), the odds of adolescents exhibiting four or more unhealthy lifestyle behaviours in medium HDI countries were higher but not statistically significant (AOR = 1.38, 95% CI: 0.91, 2.10). In contrast, countries with high HDI had significantly higher odds of this outcome (AOR = 1.84, 95% CI: 1.16, 2.94), indicating an 84% increased likelihood compared to low HDI countries. The odds were even higher in very high HDI countries, where Adolescents had three times the odds of this outcome compared to low HDI countries (AOR = 3.00, 95% CI: 1.48, 6.08) (Table 4).

## 4. Discussion

This study analysed school-based survey data selected to be nationally representative from 73 countries, encompassing 293,770 adolescents aged 12 to 17 across 5 WHO regions. The surveys provided a broad perspective on the prevalence and clustering of unhealthy lifestyle behaviours, including insufficient physical activity, inadequate fruit and vegetable consumption, frequent fast-food consumption, frequent soft drink intake, and sedentary behaviour. The results revealed the widespread engagement in unhealthy behaviours globally and the significant co-occurrence of these behaviours. It also uncovered regional and socio-economic disparities in the clustering of multiple unhealthy behaviours among adolescents.

The two most prevalent unhealthy lifestyle behaviours among adolescents were insufficient physical activity and inadequate consumption of fruits and vegetables. Insufficient physical activity showed consistently high prevalence across the World Health Organization (WHO) regions, with rates ranging from 82.9% in Africa to 89.1% in the Western Pacific. This finding aligns with the global trend reported in a previous study, which reported that over 80% of adolescents worldwide fail to meet the WHO-recommended guideline of at least 60 min of moderate-to-vigorous physical activity per day [50]. Several factors contribute to this prevalence, including rapid urbanisation, sedentary schooling environments, and limited access to safe recreational spaces, particularly in low- and middle-income countries [51,52,53]. Given the long-term consequences of physical inactivity, including increased risks of obesity, cardiovascular diseases, mental health disorders, and other non-communicable diseases (NCDs), targeted interventions are needed. Integrating physical activity into school programmes, improving urban planning to enhance access to recreational spaces, and implementing public health policies promoting active lifestyles could help mitigate these risks [54].

Similarly, inadequate consumption of fruits and vegetables is a pervasive issue, with the highest prevalence in the Americas (81.8%) and the lowest in Africa (69.5%). Fruit and vegetable consumption among adolescents is influenced by various factors, including socio-economic status, taste preferences, parental dietary habits, and availability and accessibility of fresh produce [55]. Addressing this global issue requires coordinated efforts to improve affordability, accessibility, and awareness of healthy eating. Strategies such as school-based nutrition programmes, food subsidies, and restrictions on unhealthy food marketing can help [56].

The results of this study indicate a positive correlation between age and the prevalence of multiple unhealthy lifestyle behaviours among adolescents. Notably, each additional year of age was associated with a 6% increase in the odds of exhibiting four or more clusters of unhealthy lifestyle behaviours. Consistent results were observed in previous studies [57,58]. The rise in unhealthy behaviours among older adolescents may be influenced by increased academic pressure and involvement in part-time employment [59], highlighting the need for early and sustained intervention strategies to mitigate the adoption of multiple unhealthy lifestyle behaviours

In this study, female adolescents had 16% higher odds of engaging in four or more clusters of unhealthy behaviours compared to their male counterparts. This finding is consistent with a previous study among Malaysian adolescents, which also reported a higher clustering effect of lifestyle behaviours among girls than boys [60]. This disparity highlights the importance of gender-specific interventions, mainly targeting factors such as physical inactivity, sedentary behaviour, and poor dietary habits among female adolescents.

This study found that adolescents with high levels of family support and supportive peer networks at school had significantly lower odds of engaging in four or more clusters of unhealthy lifestyle behaviours. In line with our findings, previous studies reported that adolescents with a higher perceived parental and peer support had lower odds of consuming sugary beverages, higher odds of eating fruits and vegetables, and a higher overall healthy eating score compared to those with lower perceived support [61]. Similarly, adolescents who receive strong emotional, instrumental, and informational support from their families and peers are reported to be better equipped to make healthier lifestyle choices [62]. High family support likely creates a secure and nurturing environment that promotes healthier behaviours, often through parental guidance, role modelling of positive habits, and a stable home setting. Likewise, supportive peers can contribute by providing a social network that encourages positive behaviours, offers companionship in physical activities, and supports healthier dietary choices.

Another noteworthy finding is that adolescents from food-secure households had lower odds of engaging in four or more clusters of unhealthy lifestyle behaviours. In line with the current finding, a study in the USA reported that food-insecure households bought fewer fruits, dairy, and protein foods while consuming more refined grains and had lower healthy eating index scores [63]. Similarly, a study in Spain found that children and adolescents from food-insecure households had higher odds of engaging in sedentary behaviour, consuming an unhealthy diet, and experiencing poor dietary diversity [64]. In our study, household food insecurity was a proxy for low socio-economic status, suggesting that food-insecure households may struggle to afford healthy food options. Furthermore, a recent systematic review corroborates our findings, revealing that children and adolescents from low socio-economic backgrounds are at increased risk of engaging in unhealthy behaviours, including unhealthy diets and sedentary behaviour [65].

Although unhealthy behaviours are prevalent among adolescents worldwide, significant regional disparities exist. Adolescents in the African, American, and Eastern Mediterranean regions had 49%, 78%, and 42% higher odds, respectively, of engaging in four or more unhealthy behaviours compared to those in the Western Pacific region. In contrast, no significant differences were observed in the Southeast Asia region. These disparities may be influenced by socio-economic conditions, cultural norms, and public health policy variations, highlighting the need for targeted, region-specific interventions to address unhealthy behaviours in adolescents.

Regarding the socio-economic development gradient among the countries in the study, we identified a clear pattern showing adolescents from higher Human Development Index (HDI) countries had higher odds of engaging in clusters of four or more unhealthy behaviours. These findings might be explained by the theory of epidemiologic transition, which describes how patterns of morbidity and mortality shift as countries develop. In low HDI countries, infectious diseases and undernutrition are more prevalent. As countries progress in development, these are gradually replaced by chronic and non-communicable diseases, such as obesity, cardiovascular diseases, and diabetes, which are linked to unhealthy lifestyle behaviours [66]. In the context of adolescent health behaviours, the transition from traditional dietary and physical activity patterns to more sedentary lifestyles and Western diets occurs more rapidly in higher HDI countries [67,68,69]. This suggests that adolescents may adopt unhealthy lifestyles as countries develop economically and socially, potentially due to increased access to processed foods, sedentary entertainment, and other modern conveniences [70,71]. In medium HDI countries, elevated odds of engaging in unhealthy behaviours were observed, though the difference was not statistically significant compared to low HDI countries. This suggests that these countries may be in an intermediate stage of the epidemiologic transition, where traditional lifestyle behaviours still exert some influence but are increasingly being displaced by unhealthy practises [69].

### 4.1. Policy Implications

This study underscores the need for multilevel, context-specific strategies to address the widespread and co-occurring unhealthy lifestyle behaviours among adolescents. The substantial burden of these behaviours across regions and socio-economic settings calls for interventions that target individuals, families, communities, schools, and society to support adolescents’ healthy development and reduce long-term chronic disease risks. National and regional health policies should incorporate tailored physical activity programmes for young females and culturally relevant dietary education. Policies should promote strong family bonds, peer support networks, and protective factors for healthier behaviours. To reduce socio-economic disparities, governments and health organisations should ensure affordable access to nutritious food, subsidise healthy options, and regulate unhealthy advertising for adolescents [56]. Given the variation in unhealthy behaviours by countries’ development levels, policies should be adapted to each country’s stage of development.

### 4.2. Strengths and Limitations

The strength of this study includes the analysis of global samples selected to be representative of adolescents from various countries with diverse geographical locations and socio-economic characteristics that facilitate cross-country comparisons. Additionally, the survey used a similar sampling technique, research methodology, and questionnaire across all participating countries, enhancing the comparability of the findings. The study utilised multilevel logistic regression, which accounts for variations within and between countries, improving the robustness of the findings. However, there are some limitations to consider when interpreting the findings. First, while the study utilises global survey data from multiple regions, the regional estimates may not fully represent their respective regions. Not all countries across the regions participated equally in the survey. Additionally, most of the study sample originates from low- and middle-income countries, which may limit the generalizability of the findings. Second, since the surveys were conducted within the school environment, school enrolment rates may vary across countries. This variation could result in the underrepresentation of socio-economically disadvantaged adolescents who do not attend school. Furthermore, lifestyle behaviours reported in this study were self-reported, which may be subjected to recall or social desirability bias.

## 5. Conclusions

The study highlights the significant prevalence and clustering of unhealthy lifestyle behaviours, including insufficient physical activity, sedentary behaviour, inadequate fruit and vegetable consumption, frequent soft drink intake, and fast-food consumption among adolescents worldwide. The findings demonstrate that these behaviours are widespread and show distinct patterns influenced by individual, familial, and broader societal factors. The two most prevalent unhealthy lifestyle behaviours identified among adolescents were insufficient physical activity and inadequate consumption of fruits and vegetables. The clustering of unhealthy behaviours was higher among females and older adolescents, while stronger family and peer support were associated with lower engagement in multiple unhealthy behaviours. Furthermore, adolescents in countries with higher Human Development Indexes exhibited increased odds of multiple unhealthy behaviours, underscoring the complex interplay between individual characteristics and socio-economic contexts at the country level. These results emphasise the critical need for tailored, multilevel interventions that address adolescent health’s individual, social, and structural determinants. Public health strategies should prioritise strengthening family and peer support systems while addressing regional and socio-economic disparities to mitigate the long-term risks associated with unhealthy lifestyle behaviours during adolescence. Addressing these behaviour clusters can help global health policies promote healthier lifestyles and reduce non-communicable disease burdens in adolescents.

## Figures and Tables

**Table 1:** Definitions of lifestyle behaviours assessed in study.

Lifestyle Behaviours	Self-Reported Survey Questions Used in GSHS	Responding Options	Definition of Unhealthy Behaviour
Physical activity	“During the past 7 days, on how many days were you physically active for a total of at least 60 min per day?”.	0 day; 1 day; 2 days; 3 days; 4 days; 5 days; 6 days; or 7 days.	Insufficient physical activity: <60 min/day [34].
Leisure sedentary time	“How much time do you spend during a typical or usual day sitting and watching television, playing computer games, talking with friends, or doing other sitting activities?”.	less than 1 h; 1 to 2 h; 3 to 4 h; 5 to 6 h; 7 to 8 h; More than 8 h.	Sedentary: ≥3 h/day [10,35].
Fruits and/or vegetable consumption	“During the past 30 days, how many times per day did you usually eat fruits?”.	I did not eat fruit during the past 30 days; less than one time/day; 1 time/day; 2 times/day; 3 times/day; 4 times/day; 5 or more times/day.	Insufficient consumption: <5 servings/day of fruits and/or vegetables [36,37].
Consumption of carbonated soft drinks	“During the past 30 days, how many times per day did you usually drink carbonated soft drinks?”.	I did not drink carbonated soft drinks during the past 30 days; less than one time/day; 1 time/day; 2 times/day; 3 times/day; 4 times/day; 5 or more times/day.	Frequent consumption: ≥1 time/day [10].
Consumption of fast-food	“During the past 7 days, on how many days did you eat food from a fast-food restaurant”.	0 days; 1 day; 2 days; 3 days; 4 days; 5 days; 6 days; 7 days.	Frequent consumption: ≥1 day [38].

**Table 2:** Characteristics of study population by WHO regions among adolescents aged 12–17 years across 73 countries.

Variables	Overall, *n* (%)	WHO Regions					X^2^, *p*-Value
		Africa, *n* (%)	Americas, *n* (%)	Eastern Mediterranean, *n* (%)	Southeast Asia, *n* (%)	Western Pacific, *n* (%)	
Sex							0.06
Male	139,001 (51.1)	14,249 (51.7)	51,933 (49.0)	24,863 (53.6)	14,085 (51.9)	33,871 (48.6)	
Female	152,151 (48.9)	14,558 (48.3)	57,733 (51.0)	25,260 (46.4)	17,092 (48.1)	37,508 (51.4)	
Age category							<0.0001
<15 years	133,244 (52.1)	10,706 (44.8)	49,564 (45.0)	25,356 (58.6)	16,595 (61.4)	31,023 (42.3)	
≥15 years	159,160 (47.9)	18,238 (55.2)	60,719 (55.0)	24,905 (41.4)	14,790 (38.6)	40,508 (57.7)	
Weight status							<0.0001
Overweight	55,578 (16.0)	2852 (10.8)	22,087 (29.5)	11,475 (20.3)	3120 (14)	16,044 (11.2)	
Not overweight	176,499 (84.0)	17,195 (89.2)	52,207 (70.5)	33,095 (79.7)	22,985 (86)	51,017 (88.8)	
Peer support							<0.0001
Low	156,921 (57.3)	16,673 (67.4)	59,186 (54.5)	24,223 (55.8)	17,232 (55.2)	39,607 (58.3)	
High	119,828 (42.7)	7750 (32.6)	44,630 (45.5)	25,504 (44.2)	13,973 (44.8)	27,971 (41.7)	
Family support							<0.0001
low	171,937 (63.8)	14,992 (61.9)	61,369 (59.6)	29,604 (63.9)	18,994 (60.9)	46,978 (69.8)	
High	103,740 (36.2)	9369 (38.1)	41,649 (40.4)	19,990 (36.1)	12,155 (39.1)	20,577 (30.2)	
Household food security							<0.0001
No food insecurity	274,426 (93.5)	25,790 (89.7)	106,506 (97.3)	460,89 (92.4)	29,551 (94.2)	66,490 (93.6)	
Food insecurity	17,545 (6.5)	3091 (10.3)	3536 (2.7)	4123 (7.6)	1793 (5.8)	5002 (6.4)	
World Bank Income category							
Low-income	13,536 (6.5)	3883 (10.4)	0 (0)	9653 (26.5)	0 (0)	0 (0)	<0.0001
Lower middle-income	103,350 (72.9)	15,816 (86.3)	10,477 (17.4)	18,724 (59.3)	26,121 (85.9)	32,212 (87.6)	
Upper middle-income	125,677 (17.3)	7157 (3.2)	78,799 (63.1)	1864 (9.8)	5382 (14.1)	32,475 (12.1)	
High-income	51,207 (3.3)	2307 (0.1)	21,423 (19.4)	20,382 (4.4)	0 (0)	7095 (0.3)	
Human Development Index							<0.0001
Low	23,489 (13.2)	11,801 (53)	0 (0)	11,688 (33.9)	0 (0)	0 (0)	
Medium	60,495 (23.5)	7815 (25.9)	16,375 (24.4)	10,997 (34.1)	12,313 (29.5)	12,995 (6.2)	
High	71,589 (49.1)	7240 (21.1)	16,416 (25.8)	7556 (27.6)	13,808 (56.4)	26,569 (81.7)	
Very High	138,197 (14.2)	2307 (0.1)	77,908 (49.8)	20,382 (4.4)	5382 (14.1)	32,218 (12.1)	

Percentages in table are weighted for complex survey samples.

**Table 3:** Prevalence and odds of four or more unhealthy lifestyle behaviours among adolescents aged 12–17 years across 73 countries.

Variables	Clustering of Multiple Unhealthy Lifestyle Behaviours		COR (95% CI)
	<4 Unhealthy Behaviours, *n* (%)	≥4 Unhealthy Behaviours, *n* (%)	
Sex			
Male	98,049 (74.6)	40,952 (25.4)	1.00
Female	102,016 (73.4)	50,135 (26.6)	1.15 (1.13, 1.17) ***
Age category			
<15 years	92,484 (74.8)	40,760 (25.2)	1.00
≥15 years	108,338 (73.0)	50,822 (27.0)	1.11 (1.09, 1.123) ***
Weight status			
Overweight/obese	36,631 (69.4)	18,947 (30.6)	0.99 (0.96, 1.01)
Not overweight/obese	122,855 (74.5)	53,644 (25.5)	1.00
Peer support			
Low	107,372 (73.7)	49,549 (26.3)	1.00
High	82,819 (75.2)	37,009 (24.8)	0.95 (0.93, 0.96) ***
Family support			
low	116,171 (73.5)	55,766 (26.5)	1.00
High	73,326 (76.1)	30,414 (23.9)	0.84 (0.83, 0.86) ***
Household food security			
No food insecurity	188,800 (74.0)	85,626 (26.0)	0.90 (0.87, 0.93) ***
Food insecurity	11,734 (73.2)	5811 (26.8)	1.00
WHO region			
Africa	20,976 (75.9)	8187 (24.1)	1.01 (0.67, 1.53)
Americas	72,348 (65.1)	38,351 (34.9)	2.00 (1.45, 2.76) ***
Eastern Mediterranean	33,324 (75.5)	17,299 (24.5)	1.24 (0.86, 1.81)
Southeast Asia	22,801 (74.5)	8702 (25.5)	0.91 (0.56, 1.49)
Western Pacific	52,316 (75.3)	19,466 (24.7)	1.00
World Bank Income category			
Low-income	10,405 (76.9)	3131 (23.1)	1.00
Lower middle-income	77,345 (77.2)	26,005 (22.8)	1.04 (0.71, 1.52)
Upper middle-income	86,283 (62.0)	39,394 (38.0)	1.90 (1.29, 2.80) **
High-income	27,732 (58.9)	23,475 (41.1)	2.94 (1.99, 4.32) ***
Human Development Index			
Low	18,885 (82.4)	4604 (17.6)	1.00
Medium	44,381 (76.4)	16,114 (23.6)	1.31 (0.92, 1.88)
High	49,094 (74.6)	22,495 (25.4)	1.93 (1.35, 2.75) ***
Very High	89,405 (59.9)	48,792 (40.1)	3.16 (2.24, 4.45) ***

Percentages in table weighted for complex survey sample. COR: Crude Odds Ratio; CI: confidence interval. ** Significant at *p* < 0.01; *** significant at *p* < 0.0001.

**Table 4:** Multivariable multilevel logistic regression analysis of factors associated with exhibiting four or more unhealthy lifestyle behaviours among adolescents aged 12–17 across 73 countries.

Variables	Model I	Model II	Model III
	AOR (95% CI)	AOR (95% CI)	AOR (95% CI)
Age	-	1.06 (1.05, 1.07) ***	1.06 (1.05, 1.07) ***
Sex			
Male	-	1.00	1.00
Female	-	1.16 (1.14, 1.19) ***	1.16 (1.14, 1.19) ***
Weight status			
Overweight/obese	-	1.00 (0.98, 1.03)	1.00 (0.98, 1.03)
Not overweight/obese	-	1.00	1.00
Peer support			
low	-	1.00	1.00
High	-	0.96 (0.94, 0.98) ***	0.96 (0.94, 0.98) ***
Family support			
Low	-	1.00	1.00
High	-	0.84 (0.82, 0.86) ***	0.84 (0.82, 0.86) ***
Household food security			
No food insecurity	-	0.91 (0.87, 0.95) ***	0.91 (0.87, 0.95) ***
Food insecurity	-	1.00	1.00
WHO regions			
Western Pacific	-	-	1.00
Africa	-	-	1.49 (1.04, 2.15) *
Americas	-	-	1.78 (1.37, 2.30) ***
Eastern Mediterranean	-	-	1.42 (1.06, 1.90) *
Southeast Asia	-	-	1.14 (0.78, 1.65)
World Bank Income category			
Low-income	-	-	1.00
Lower middle-income	-	-	1.04 (0.64, 1.69)
Upper middle-income	-	-	1.19 (0.68, 2.09)
High-income	-	-	1.07 (0.50, 2.32)
Human Development Index			
Low	-	-	1.00
Medium	-	-	1.38 (0.91, 2.10)
High	-	-	1.84 (1.16, 2.94) *
Very High	-	-	3.00 (1.48, 6.08) *
Measures of variation			
Country-level variance (SE)	0.38 (0.062) ***	0.36 (0.067) ***	0.12 (0.022) ***
Explained variation-PCV (%)	Reference	5.26	68.42
MOR	2.04 (1.83, 2.31)	2.01 (1.79, 2.31)	1.49 (1.39, 1.61)
ICC (%)	10.2	9.8	3.4
Model fit statistics			
Deviance	349,648	223,540	223,475
Log-likelihood	−174,824	−111,770	−111,737
AIC	349,652	223,556	223,515

Model I: intercept only model, model II: adjusted for age, sex, weight status, peer support, family support, household food insecurity; model III: adjusted for variables in model II, WHO regions, World Bank Income category, Human Development Index, sample size, and years of surveys; AOR, adjusted odds ratio; MOR, median odds ratio; ICC, intra-class correlation; PCV, percentage change in variance. * Significant at *p* < 0.05; *** significant at *p* < 0.0001.

## Data Availability

This study utilised publicly accessible data from the Global School-based Student Health Survey (GSHS), an ongoing international collaborative surveillance initiative jointly conducted by the World Health Organization (WHO) and the U.S. Centers for Disease Control and Prevention (CDC). The original data presented in the study are openly available at the following link: https://www.who.int/teams/noncommunicable-diseases/surveillance/systems-tools/global-school-based-student-health-survey (accessed on 6 November 2023).

## Supplementary Materials

The following supporting information can be downloaded at: https://www.mdpi.com/article/10.3390/nu17040609/s1. Table S1: Characteristics of survey samples included in study by WHO regions among adolescents aged 12–17 years across 73 countries. Table S2: Prevalence of unhealthy behaviours among adolescents by countries of survey among adolescents aged 12–17 years across 73 countries. Table S3: Prevalence of the co-occurring unhealthy lifestyle behaviour among adolescents aged 12–17 years across 73 countries. Table S4: Prevalence of unhealthy lifestyle behaviours patterns among adolescents aged 12–17 years across 73 countries.
