# Supplementary material for: Clustering Patterns of Unhealthy Lifestyle Behaviours Among Adolescents: A Multilevel Analysis of a Nationally Representative School-Based Survey from 73 Countries
Source: Nutrients. 2025 Feb 7;17(4):609. doi: 10.3390/nu17040609 (PMC11857990; doi:10.3390/nu17040609)
Supplement: Supplementary file 1 [file nutrients-17-00609-s001.zip › nutrients-3392743-supplementary.pdf]

**Clustering Patterns of Unhealthy Lifestyle Behaviours Among Adolescents: A Multilevel Analysis of a Nationally Representative School-Based Survey from 73 Countries**

## Contents

Table S1: Characteristics of survey samples included in the study by WHO regions among adolescents aged 12-17 years across 73 countries. ....................................................................2

Table S2: Prevalence of unhealthy behaviours among adolescents by countries of survey among adolescents aged 12-17 years across 73 countries. .......................................................4

Table S3: Prevalence of the co-occurring unhealthy lifestyle behaviour among adolescents among adolescents aged 12-17 years across 73 countries. .......................................................7

Table S4: Prevalence of unhealthy lifestyle behaviours patterns among adolescents among adolescents aged 12-17 years across 73 countries. .................................................................10

**Table S1: Characteristics of survey samples included in the study by WHO regions among adolescents aged 12-17 years across 73 countries.**

| Countries | Year | Sample size | Male (%) | Female (%) | Response rate |
|---|---|---|---|---|---|
| **Africa** | | | | | |
| Algeria | 2011 | 4386 | 48.1 | 51.9 | 98 |
| Benin | 2016 | 2482 | 73.0 | 27.0 | 78 |
| Ghana | 2012 | 3512 | 51.4 | 48.6 | 76 |
| Liberia | 2017 | 2161 | 51.8 | 48.2 | 71 |
| Mauritania | 2010 | 1856 | 54.5 | 45.5 | 70 |
| Mauritius | 2017 | 2854 | 46.5 | 53.5 | 84 |
| Mozambique | 2015 | 1722 | 52.9 | 47.1 | 80 |
| Namibia | 2013 | 4303 | 46.9 | 53.1 | 89 |
| Seychelles | 2015 | 2307 | 50.4 | 49.6 | 82 |
| United Republic of Tanzania | 2014 | 3580 | 49.1 | 50.9 | 87 |
| **Americas** | | | | | |
| Anguilla | 2016 | 748 | 49.3 | 50.7 | 88 |
| Antigua and Barbuda | 2009 | 1136 | 51.6 | 48.4 | 67 |
| Argentina | 2018 | 53865 | 48.0 | 52.0 | 63 |
| Bahamas | 2013 | 1228 | 48.0 | 52.0 | 78 |
| Barbados | 2011 | 1477 | 49.4 | 50.6 | 73 |
| Belize | 2011 | 1957 | 48.2 | 51.8 | 88 |
| Bolivia | 2018 | 6974 | 50.7 | 49.3 | 79 |
| British Virgin Islands | 2009 | 1558 | 47.5 | 52.5 | 90 |
| Chile | 2013 | 1925 | 49.7 | 50.4 | 60 |
| Costa Rica | 2009 | 2620 | 50.4 | 49.6 | 72 |
| Curaçao | 2015 | 2502 | 48.9 | 51.1 | 83 |
| Dominican Republic | 2016 | 1347 | 49.6 | 50.4 | 63 |
| El Salvador | 2013 | 1837 | 51.5 | 48.5 | 88 |
| Guatemala | 2015 | 3863 | 52.4 | 47.6 | 82 |
| Guyana | 2009 | 2238 | 48.6 | 51.5 | 76 |
| Honduras | 2012 | 1666 | 46.9 | 53.2 | 79 |
| Jamaica | 2017 | 1513 | 49.2 | 50.9 | 60 |
| Panama | 2018 | 2815 | 47.4 | 52.7 | 71 |
| Paraguay | 2017 | 2924 | 48.8 | 51.2 | 87 |
| Peru | 2010 | 2848 | 50.6 | 49.4 | 85 |
| Saint Kitts and Nevis | 2010 | 1635 | 51.3 | 48.7 | 70 |
| Saint Lucia | 2018 | 1828 | 48.0 | 52.0 | 77 |
| Saint Vincent and the Grenadines | 2018 | 1761 | 48.9 | 51.1 | 78 |
| Suriname | 2016 | 2035 | 49.1 | 50.9 | 83 |
| Trinidad and Tobago | 2017 | 3511 | 48.2 | 51.9 | 89 |
| Uruguay | 2019 | 2888 | 45.2 | 54.8 | 64 |
| **Eastern Mediterranean** | | | | | |
| Afghanistan | 2014 | 2281 | 55.3 | 44.7 | 79 |

| | | | | | |
|---|---|---|---|---|---|
| Bahrain | 2016 | 6947 | 51.1 | 48.9 | 89 |
| Egypt | 2011 | 2377 | 50.3 | 49.7 | 85 |
| Iraq | 2012 | 1864 | 57.3 | 42.7 | 88 |
| Kuwait | 2015 | 3107 | 48.9 | 51.1 | 78 |
| Lebanon | 2017 | 5179 | 46.8 | 53.2 | 82 |
| Morocco | 2016 | 6162 | 53.8 | 46.2 | 91 |
| Oman | 2015 | 3256 | 49.8 | 50.2 | 92 |
| Pakistan | 2009 | 5006 | 61.2 | 38.8 | 76 |
| Qatar | 2011 | 1578 | 48.2 | 51.8 | 87 |
| Sudan | 2012 | 2008 | 52.5 | 47.5 | 77 |
| Syrian Arab Republic | 2010 | 2971 | 51.2 | 48.8 | 97 |
| United Arab Emirates | 2016 | 5494 | 49.7 | 50.3 | 80 |
| Yemen | 2014 | 2393 | 55.3 | 44.7 | 75 |
| **South-East Asia** | | | | | |
| Bangladesh | 2014 | 2734 | 65.2 | 34.8 | 91 |
| Indonesia | 2015 | 10637 | 48.9 | 51.1 | 94 |
| Nepal | 2015 | 6153 | 48.7 | 51.3 | 69 |
| Sri Lanka | 2016 | 3171 | 48.9 | 51.1 | 89 |
| Thailand | 2021 | 5382 | 46.9 | 53.1 | 83 |
| Timor-Leste | 2015 | 3426 | 50.7 | 49.3 | 79 |
| **Western Pacific** | | | | | |
| Brunei | 2019 | 2373 | 50.5 | 49.5 | 62 |
| Cambodia | 2013 | 3705 | 52.3 | 47.7 | 85 |
| Cook Islands | 2015 | 689 | 48.6 | 51.4 | 65 |
| Fiji | 2016 | 3337 | 49.2 | 50.8 | 79 |
| French Polynesia | 2015 | 3036 | 49.5 | 50.5 | 70 |
| Kiribati | 2011 | 1529 | 47.2 | 52.8 | 85 |
| Lao People's Democratic Republic | 2015 | 3603 | 53.3 | 46.7 | 70 |
| Malaysia | 2012 | 25123 | 50.2 | 49.8 | 89 |
| Mongolia | 2013 | 5217 | 48.3 | 51.7 | 88 |
| Philippines | 2019 | 9845 | 49.5 | 50.5 | 85 |
| Samoa | 2017 | 1748 | 47.8 | 52.2 | 59 |
| Solomon Islands | 2011 | 1304 | 54.2 | 45.8 | 85 |
| Tonga | 2017 | 3169 | 50.7 | 49.3 | 90 |
| Tuvalu | 2013 | 846 | 48.4 | 51.6 | 90 |
| Vanuatu | 2016 | 2008 | 49.6 | 50.4 | 57 |
| Vietnam | 2013 | 3253 | 46.9 | 53.1 | 96 |
| Wallis and Futuna | 2015 | 997 | 48.9 | 51.1 | 82 |

**Footnotes:** Percentages in the table are weighted for complex survey samples.

**Table S2: Prevalence of unhealthy behaviours among adolescents by countries of survey among adolescents aged 12-17 years across 73 countries.**

| Countries | Insufficient physical activity | Sedentary behaviour | Insufficient consumption of FV | Soft drink consumption | Fast food consumption |
|---|---|---|---|---|---|
| | Percent | Percent | Percent | Percent | Percent |
| **Africa** | | | | | |
| Regional average | 82.9 | 23.6 | 69.6 | 51.8 | 48.5 |
| Algeria | 84.4 | 27.3 | 66.1 | 76.9 | 52.3 |
| Benin | 70.3 | 23.1 | 74.4 | 34.8 | 38.9 |
| Ghana | 87.5 | 19.4 | 71.3 | 45.4 | 64.0 |
| Liberia | 87.7 | 19.2 | 70.1 | 45.1 | 40.2 |
| Mauritania | 87.8 | 37.2 | 70.8 | 50.1 | 62.0 |
| Mauritius | 81.1 | 40.4 | 74.7 | 40.6 | 57.8 |
| Mozambique | 85.9 | 37.1 | 78.8 | 56.1 | 60.1 |
| Namibia | 85.7 | 35.7 | 78.2 | 46.5 | 48.8 |
| Seychelles | 82.1 | 51.1 | 60.8 | 68.2 | 69.5 |
| United Republic of Tanzania | 80.2 | 19.9 | 66.4 | 44.9 | 35.1 |
| **Americas** | | | | | |
| Regional average | 85.1 | 43.4 | 81.8 | 50.4 | 47.2 |
| Anguilla | 81.3 | 59.7 | 82.7 | 52.4 | 75.4 |
| Antigua and Barbuda | 76.7 | 55.2 | 73.5 | 58.3 | 56.5 |
| Argentina | 83.5 | 55.5 | 87.2 | 32.8 | 39.4 |
| Bahamas | 84.8 | 54.4 | 84.7 | 68.8 | 71.9 |
| Barbados | 81.4 | 66.2 | 87.5 | 73.4 | 62.4 |
| Belize | 79.2 | 37.5 | 70.1 | 64.0 | 65.1 |
| Bolivia | 88.7 | 31.6 | 78.1 | 33.9 | 62.2 |
| British Virgin Islands | 82.5 | 61.7 | 85.7 | 64.0 | 55.6 |
| Chile | 86.0 | 53.8 | 74.0 | 65.2 | 34.7 |
| Costa Rica | 81.5 | 43.7 | 81.5 | 51.7 | 53.8 |
| Curaçao | 88.1 | 62.6 | 83.8 | 60.1 | 69.0 |
| Dominican Republic | 87.7 | 47.1 | 83.4 | 73.1 | 48.6 |
| El Salvador | 87.2 | 34.4 | 79.1 | 66.5 | 56.8 |
| Guatemala | 89.1 | 21.9 | 72.0 | 61.4 | 57.7 |
| Guyana | 84.6 | 36.3 | 68.7 | 70.7 | 54.9 |
| Honduras | 84.3 | 30.3 | 74.2 | 73.8 | 46.3 |
| Jamaica | 76.6 | 56.9 | 82.6 | 68.6 | 58.4 |
| Panama | 85.3 | 48.1 | 89.7 | 34.6 | 53.8 |

| | | | | | |
|---|---|---|---|---|---|
| Paraguay | 83.3 | 34.3 | 74.3 | 59.7 | 54.2 |
| Peru | 84.7 | 28.8 | 90.8 | 53.9 | 50.3 |
| Saint Kitts and Nevis | 81.4 | 59.0 | 84.3 | 61.2 | 60.1 |
| Saint Lucia | 78.9 | 56.3 | 76.9 | 52.9 | 62.1 |
| Saint Vincent and the Grenadines | 81.7 | 54.9 | 90.8 | 41.1 | 61.4 |
| Suriname | 81.4 | 42.5 | 70.3 | 79.0 | 63.0 |
| Trinidad and Tobago | 79.8 | 48.6 | 82.8 | 52.2 | 68.7 |
| Uruguay | 86.6 | 63.0 | 87.7 | 23.4 | 36.1 |
| **Eastern Mediterranean** | | | | | |
| Regional average | 87.7 | 25.3 | 78.4 | 42.1 | 47.5 |
| Afghanistan | 90.1 | 24.4 | 84.7 | 37.6 | 65.1 |
| Bahrain | 80.1 | 58.4 | 78.9 | 34.4 | 74.2 |
| Egypt | 86.6 | 27.4 | 76.3 | 54.5 | 49.1 |
| Iraq | 85.1 | 26.0 | 74.6 | 53.5 | 56.0 |
| Kuwait | 84.6 | 65.2 | 84.6 | 53.2 | 75.8 |
| Lebanon | 85.6 | 44.9 | 78.4 | 47.4 | 77.4 |
| Morocco | 89.3 | 32.4 | 67.1 | 32.0 | 64.1 |
| Oman | 88.7 | 39.9 | 78.7 | 43.2 | 71.4 |
| Pakistan | 88.3 | 8.2 | 90.0 | 36.1 | 21.0 |
| Qatar | 89.4 | 48.2 | 74.6 | 60.5 | 85.8 |
| Sudan | 91.9 | 18.9 | 78.7 | 37.8 | 38.7 |
| Syrian Arab Republic | 89.0 | 25.6 | 84.8 | 30.6 | 42.3 |
| United Arab Emirates | 84.3 | 59.3 | 78.0 | 34.1 | 76.9 |
| Yemen | 87.6 | 21.8 | 79.4 | 37.1 | 37.6 |
| **South-East Asia** | | | | | |
| Regional average | 81.5 | 29.4 | 80.9 | 33.0 | 58.8 |
| Bangladesh | 58.3 | 15.4 | 84.0 | 47.3 | 52.7 |
| Indonesia | 87.7 | 27.4 | 76.8 | 27.7 | 54.2 |
| Nepal | 84.7 | 10.6 | 90.7 | 32.6 | 74.8 |
| Sri Lanka | 84.5 | 37.3 | 76.8 | 26.1 | 41.7 |
| Thailand | 89.3 | 65.2 | 86.7 | 34.5 | 80.6 |
| Timor-Leste | 90.3 | 14.7 | 84.5 | 42.2 | 65.1 |
| **Western Pacific** | | | | | |
| Regional average | 89.1 | 37.8 | 81.4 | 31.6 | 43.2 |
| Brunei | 88.1 | 56.6 | 79.9 | 40.2 | 67.1 |
| Cambodia | 92.5 | 12.0 | 90.1 | 41.8 | 21.9 |
| Cook Islands | 84.8 | 44.1 | 65.9 | 55.3 | 67.8 |
| Fiji | 79.0 | 29.1 | 65.4 | 62.1 | 61.3 |
| French Polynesia | 82.0 | 40.7 | 68.6 | 45.9 | 71.6 |
| Kiribati | 82.3 | 15.2 | 85.0 | 21.9 | 43.7 |

| | | | | | |
|---|---|---|---|---|---|
| Lao People's Democratic Republic | 83.4 | 20.9 | 83.6 | 50.3 | 41.6 |
| Malaysia | 85.9 | 47.4 | 71.4 | 29.2 | 46.9 |
| Mongolia | 74.8 | 44.7 | 81.3 | 33.4 | 55.1 |
| Philippines | 93.6 | 34.2 | 85.2 | 31.4 | 56.9 |
| Samoa | 77.3 | 27.5 | 58.5 | 63.6 | 67.5 |
| Solomon Islands | 83.4 | 25.4 | 57.0 | 44.3 | 66.6 |
| Tonga | 82.3 | 21.0 | 56.9 | 59.7 | 69.4 |
| Tuvalu | 87.9 | 17.3 | 68.6 | 52.0 | 42.8 |
| Vanuatu | 87.4 | 20.3 | 50.4 | 38.8 | 54.4 |
| Vietnam | 86.0 | 41.9 | 79.8 | 30.0 | 30.0 |
| Wallis and Futuna | 86.8 | 37.9 | 80.0 | 56.5 | 64.4 |

**Footnotes:** Percentages in the table are weighted for complex survey samples.

**Table S3: Prevalence of the co-occurring unhealthy lifestyle behaviour among adolescents among adolescents aged 12-17 years across 73 countries.**

| Countries | Prevalence of the number of co-occurring risk behaviours (%) [a] | | | | | | Mean (SE) |
|---|---|---|---|---|---|---|---|
| | 0 | 1 | 2 | 3 | 4 | 5 | |
| **Africa** | | | | | | | |
| Regional average | 1.0 | 9.0 | 30.8 | 35.1 | 20.1 | 4.0 | 2.76 (0.03) |
| Algeria | 0.2 | 4.9 | 22.8 | 38.0 | 28.3 | 5.9 | 3.07 (0.04) |
| Benin | 1.4 | 15.8 | 39.1 | 29.3 | 12.4 | 2.0 | 2.41 (0.05) |
| Ghana | 0.5 | 5.9 | 28.3 | 39.8 | 21.9 | 3.6 | 2.88 (0.04) |
| Liberia | 1.6 | 9.8 | 35.3 | 33.9 | 17.2 | 2.3 | 2.62 (0.04) |
| Mauritania | 0.4 | 5.0 | 22.2 | 38.3 | 27.1 | 7.1 | 3.08 (0.04) |
| Mauritius | 1.7 | 8.5 | 24.8 | 31.9 | 24.8 | 8.3 | 2.95 (0.04) |
| Mozambique | 0.1 | 4.6 | 20.3 | 36.4 | 29.5 | 9.1 | 3.18 (0.05) |
| Namibia | 0.4 | 5.5 | 28.8 | 35.4 | 24.0 | 5.9 | 2.95 (0.04) |
| Seychelles | 1.1 | 4.4 | 15.7 | 32.8 | 32.5 | 13.5 | 3.32 (0.04) |
| United Republic of Tanzania | 2.1 | 13.3 | 38.3 | 31.2 | 12.9 | 2.3 | 2.46 (0.04) |
| **Americas** | | | | | | | |
| Regional average | 0.6 | 5.3 | 22.6 | 36.6 | 26.9 | 8.1 | 3.08 (0.01) |
| Anguilla | 0.3 | 3.6 | 16.1 | 26.0 | 32.7 | 21.4 | 3.52 (0.04) |
| Antigua and Barbuda | 0.5 | 5.2 | 20.3 | 32.7 | 30.4 | 11.0 | 3.20 (0.05) |
| Argentina | 0.7 | 5.9 | 24.3 | 38.9 | 24.2 | 6.1 | 2.98 (0.01) |
| Bahamas | 0.3 | 2.2 | 11.6 | 26.1 | 38.5 | 21.4 | 3.65 (0.05) |
| Barbados | 0.1 | 1.9 | 11.5 | 24.6 | 37.5 | 24.5 | 3.71 (0.03) |
| Belize | 0.6 | 6.3 | 20.2 | 33.8 | 28.2 | 11.0 | 3.16 (0.05) |
| Bolivia | 0.7 | 6.3 | 25.4 | 38.6 | 23.8 | 5.3 | 2.94 (0.02) |
| British Virgin Islands | 0.2 | 3.1 | 14.0 | 30.5 | 34.3 | 18.0 | 3.49 (6.93e-15) |
| Chile | 0.7 | 5.6 | 21.1 | 33.9 | 29.3 | 9.4 | 3.14 (0.03) |
| Costa Rica | 0.7 | 5.7 | 22.1 | 33.7 | 27.7 | 10.0 | 3.12 (0.03) |
| Curaçao | 0.3 | 2.5 | 10.9 | 28.6 | 35.3 | 22.5 | 3.64 (0.03) |
| Dominican Republic | 0.4 | 2.5 | 14.4 | 35.8 | 33.5 | 13.4 | 3.40 (0.05) |
| El Salvador | 0.8 | 4.1 | 19.8 | 32.1 | 31.9 | 11.2 | 3.24 (0.05) |
| Guatemala | 1.2 | 5.3 | 22.9 | 38.2 | 25.5 | 6.9 | 3.02 (0.05) |
| Guyana | 0.5 | 5.1 | 20.0 | 36.6 | 29.0 | 8.8 | 3.15 (0.03) |
| Honduras | 0.5 | 4.6 | 22.4 | 37.8 | 27.5 | 7.3 | 3.09 (0.04) |
| Jamaica | 0.3 | 2.7 | 13.8 | 34.3 | 34.6 | 14.3 | 3.43 (0.04) |
| Panama | 0.5 | 4.6 | 22.3 | 36.0 | 28.7 | 7.9 | 3.12 (0.05) |
| Paraguay | 0.9 | 6.1 | 23.7 | 33.8 | 26.3 | 9.2 | 3.06 (0.04) |
| Peru | 0.2 | 4.9 | 24.3 | 35.6 | 26.8 | 8.2 | 3.08 (0.04) |
| Saint Kitts and Nevis | 0.2 | 3.4 | 14.5 | 30.4 | 34.9 | 16.6 | 3.46 (1.92e-14) |

| | | | | | | | |
|---|---|---|---|---|---|---|---|
| Saint Lucia | 0.8 | 6.2 | 16.0 | 31.7 | 32.8 | 12.6 | 3.27 (0.03) |
| Saint Vincent and the Grenadines | 0.4 | 3.2 | 18.2 | 33.4 | 34.5 | 10.5 | 3.30 (0.03) |
| Suriname | 0.3 | 4.1 | 15.6 | 33.0 | 33.0 | 14.0 | 3.36 (0.03) |
| Trinidad and Tobago | 0.8 | 5.0 | 15.8 | 31.9 | 32.5 | 14.0 | 3.32 (0.03) |
| Uruguay | 0.5 | 6.4 | 22.6 | 42.2 | 22.6 | 5.6 | 2.97 (0.03) |
| **Eastern Mediterranean** | | | | | | | |
| Regional average | 0.5 | 6.4 | 33.7 | 34.9 | 19.9 | 4.6 | 2.81 (0.02) |
| Afghanistan | 0.2 | 3.6 | 25.5 | 40.7 | 25.0 | 5.1 | 3.02 (0.06) |
| Bahrain | 1.0 | 5.7 | 17.1 | 30.8 | 33.3 | 12.1 | 3.26 (0.03) |
| Egypt | 0.5 | 4.6 | 28.7 | 38.7 | 21.9 | 5.7 | 2.94 (0.06) |
| Iraq | 0.4 | 5.6 | 27.3 | 36.5 | 25.5 | 4.7 | 2.95 (0.03) |
| Kuwait | 0.5 | 3.7 | 11.4 | 24.7 | 36.0 | 23.8 | 3.63 (0.06) |
| Lebanon | 0.5 | 3.8 | 16.4 | 33.6 | 32.3 | 13.5 | 3.34 (0.03) |
| Morocco | 0.8 | 7.5 | 29.6 | 35.3 | 21.6 | 5.2 | 2.85 (0.04) |
| Oman | 0.5 | 4.8 | 19.6 | 34.3 | 29.2 | 11.6 | 3.22 (0.04) |
| Pakistan | 0.3 | 8.6 | 50.1 | 29.8 | 10.5 | 0.7 | 2.44 (0.05) |
| Qatar | 0.3 | 2.8 | 10.9 | 29.7 | 37.1 | 19.3 | 3.58 (0.04) |
| Sudan | 0.4 | 7.6 | 39.7 | 33.1 | 16.3 | 2.9 | 2.66 (0.04) |
| Syrian Arab Republic | 0.5 | 6.5 | 36.9 | 36.0 | 16.9 | 3.3 | 2.72 (0.05) |
| United Arab Emirates | 0.6 | 4.4 | 16.3 | 31.9 | 34.2 | 12.6 | 3.33 (0.04) |
| Yemen | 0.6 | 7.7 | 40.8 | 32.0 | 16.4 | 2.5 | 2.63 (0.07) |
| **South-East Asia** | | | | | | | |
| Regional average | 0.6 | 7.2 | 29.7 | 37.0 | 21.5 | 3.9 | 2.84 (0.02) |
| Bangladesh | 0.7 | 10.3 | 38.4 | 34.4 | 13.7 | 2.6 | 2.58 (0.04) |
| Indonesia | 0.7 | 7.7 | 32.4 | 38.2 | 18.7 | 2.4 | 2.74 (0.03) |
| Nepal | 0.1 | 4.9 | 23.4 | 47.0 | 22.4 | 2.3 | 2.93 (0.04) |
| Sri Lanka | 1.4 | 8.7 | 34.9 | 35.5 | 16.3 | 3.2 | 2.66 (0.04) |
| Thailand | 0.0 | 1.7 | 10.0 | 31.2 | 44.4 | 12.6 | 3.56 (0.03) |
| Timor-Leste | 0.2 | 4.7 | 24.8 | 41.9 | 25.3 | 3.1 | 2.97 (0.02) |
| **Western Pacific** | | | | | | | |
| Regional average | 0.5 | 6.8 | 30.3 | 37.7 | 20.8 | 3.9 | 2.83 (0.02) |
| Brunei | 0.4 | 3.5 | 16.6 | 34.6 | 33.3 | 11.6 | 3.32 (0.03) |
| Cambodia | 0.2 | 5.3 | 45.9 | 34.8 | 12.5 | 1.4 | 2.58 (0.04) |
| Cook Islands | 0.9 | 6.9 | 21.1 | 29.3 | 28.0 | 13.7 | 3.18 (0.06) |
| Fiji | 1.1 | 8.0 | 23.1 | 35.4 | 25.4 | 7.0 | 2.97 (0.05) |
| French Polynesia | 1.3 | 6.3 | 21.8 | 33.3 | 27.7 | 9.7 | 3.09 (0.03) |
| Kiribati | 1.0 | 12.1 | 39.6 | 34.1 | 12.0 | 1.3 | 2.48 (0.04) |
| Lao People's Democratic Republic | 0.5 | 7.3 | 32.4 | 35.4 | 20.8 | 3.6 | 2.80 (0.05) |
| Malaysia | 1.1 | 8.8 | 28.1 | 36.8 | 20.9 | 4.4 | 2.81 (0.02) |
| Mongolia | 1.0 | 8.3 | 27.4 | 33.6 | 22.9 | 6.8 | 2.89 (0.05) |
| Philippines | 0.2 | 3.6 | 26.3 | 39.1 | 26.0 | 4.8 | 3.01 (0.03) |
| Samoa | 2.0 | 7.6 | 22.6 | 36.3 | 24.8 | 6.8 | 2.94 (0.04) |
| Solomon Islands | 1.0 | 10.3 | 27.7 | 36.7 | 20.9 | 3.5 | 2.77 (0.05) |

| | | | | | | | |
|---|---|---|---|---|---|---|---|
| Tonga | 1.5 | 8.5 | 23.8 | 36.2 | 25.3 | 4.7 | 2.89 (0.03) |
| Tuvalu | 0.7 | 7.6 | 34.9 | 38.4 | 16.3 | 2.2 | 2.68 (1.97e-15) |
| Vanuatu | 1.2 | 14.7 | 33.5 | 34.6 | 14.0 | 1.9 | 2.51 (0.04) |
| Vietnam | 0.7 | 9.5 | 33.3 | 37.2 | 16.4 | 2.9 | 2.68 (0.03) |
| Wallis and Futuna | 0.4 | 3.9 | 20.6 | 32.3 | 30.6 | 12.3 | 3.26 (0.04) |

**Footnotes:** Percentages in the table are weighted for complex survey samples.

**Table S4: Prevalence of unhealthy lifestyle behaviours patterns among adolescents among adolescents aged 12-17 years across 73 countries.**

| S. N | Number of risk factors | Pi | Sb | FVi | Soft | FF | Overall | Boys | Girls |
|---|---|---|---|---|---|---|---|---|---|
| | | | | | | | Percent (95% CI) | Percent (95% CI) | Percent (95% CI) |
| 1 | 0 | - | - | - | - | - | 0.6 (0.5, 0.7) | 0.8 (0.6, 0.9) | 0.5 (0.4, 0.5) |
| 2 | 1 | - | - | + | - | - | 2.9 (2.6, 3.1) | 3.4 (3.0, 3.7) | 2.4 (2.1, 2.7) |
| 3 | 1 | - | - | - | - | + | 0.5 (0.4, 0.6) | 0.6 (0.5, 0.7) | 0.4 (0.4, 0.5) |
| 4 | 1 | - | - | - | + | - | 0.5 (0.4, 0.6) | 0.6 (0.5, 0.7) | 0.4 (0.3, 0.4) |
| 5 | 1 | - | + | - | - | - | 0.3 (0.3, 0.4) | 0.4 (0.3, 0.5) | 0.3 (0.2, 0.3) |
| 6 | 1 | + | - | - | - | - | 2.8 (2.6, 2.9) | 2.5 (2.3, 2.7) | 3.0 (2.8, 3.3) |
| 7 | 2 | - | - | + | - | + | 1.8 (1.7, 2.0) | 2.1 (1.9, 2.3) | 1.5 (1.3, 1.7) |
| 8 | 2 | - | - | + | + | - | 1.0 (0.9, 1.2) | 1.2 (1.1, 1.4) | 0.8 (0.7, 1.0) |
| 9 | 2 | - | - | - | + | + | 1.2 (1.0, 1.4) | 1.4 (1.1, 1.7) | 1.0 (0.7, 1.3) |
| 10 | 2 | - | + | + | - | - | 1.2 (1.1, 1.3) | 1.4 (1.3, 1.6) | 0.9 (0.8, 1.0) |
| 11 | 2 | - | + | - | - | + | 0.3 (0.2, 0.3) | 0.3 (0.3, 0.4) | 0.2 (0.2, 0.3) |
| 12 | 2 | - | + | - | + | - | 0.3 (0.2, 0.3) | 0.3 (0.2, 0.4) | 0.2 (0.2, 0.3) |
| 13 | 2 | + | - | + | - | - | 18.3 (17.6, 19.0) | 18.2 (17.3, 19.1) | 18.4 (17.6, 19.3) |
| 14 | 2 | + | - | - | - | + | 2.6 (2.4, 2.8) | 2.5 (2.3, 2.7) | 2.6 (2.4, 2.9) |
| 15 | 2 | + | - | - | + | - | 2.3 (2.2, 2.5) | 2.1 (2.0, 2.3) | 2.5 (2.3, 2.8) |
| 16 | 2 | + | + | - | - | - | 1.1 (1.0, 1.2) | 1.0 (0.9, 1.1) | 1.3 (1.1, 1.4) |
| 17 | 3 | - | - | + | + | + | 1.5 (1.4, 1.7) | 2.1 (1.7, 2.4) | 1.0 (0.9, 1.2) |
| 18 | 3 | - | + | + | - | + | 1.0 (0.9, 1.1) | 1.2 (1.1, 1.3) | 0.8 (0.7, 0.9) |
| 19 | 3 | - | + | + | + | - | 0.5 (0.4, 0.5) | 0.6 (0.6, 0.8) | 0.3 (0.3, 0.4) |
| 20 | 3 | - | + | - | + | + | 0.5 (0.4, 0.6) | 0.7 (0.6, 0.8) | 0.3 (0.3, 0.4) |
| 21 | 3 | + | - | + | - | + | 12.4 (11.8, 12.9) | 12.2 (11.6, 12.7) | 12.5 (11.9, 13.2) |
| 22 | 3 | + | - | + | + | - | 7.4 (6.9, 7.8) | 7.0 (6.5, 7.5) | 7.8 (7.1, 8.5) |
| 23 | 3 | + | - | - | + | + | 3.8 (3.6, 4.1) | 3.6 (3.3, 3.9) | 4.1 (3.8, 4.4) |
| 24 | 3 | + | + | + | - | - | 7.4 (6.9, 7.8) | 6.9 (6.3, 7.4) | 7.9 (7.3, 8.4) |
| 25 | 3 | + | + | - | - | + | 1.3 (1.2, 1.4) | 1.1 (1.0, 1.2) | 1.5 (1.3, 1.6) |
| 26 | 3 | + | + | - | + | - | 0.9 (0.8, 1.0) | 0.9 (0.8, 1.0) | 0.9 (0.8, 1.0) |
| 27 | 4 | - | + | + | + | + | 0.8 (0.7, 1.0) | 1.1 (0.9, 1.3) | 0.5 (0.4, 0.6) |
| 28 | 4 | + | - | + | + | + | 8.9 (8.5, 9.3) | 9.0 (8.6, 9.5) | 8.7 (8.3, 9.2) |
| 29 | 4 | + | + | + | - | + | 7.1 (6.5, 7.7) | 6.1 (5.6, 6.7) | 8.1 (7.3, 8.9) |
| 30 | 4 | + | + | + | + | - | 2.8 (2.6, 3.0) | 2.8 (2.6, 3.1) | 2.8 (2.6, 3.0) |
| 31 | 4 | + | + | - | + | + | 1.9 (1.8, 2.0) | 1.8 (1.6, 2.0) | 2.0 (1.8, 2.2) |
| 32 | 5 | + | + | + | + | + | 4.5 (4.3, 4.8) | 4.5 (4.2, 4.9) | 4.6 (4.3, 4.9) |

**Footnotes: Pi**: Insufficient physical activity; **FVi**: Insufficient consumption of fruit and vegetable; **FF**: Fast food consumption; **Soft**: Soft drink consumption; **Sb**: Sedentary behaviour; Percentages in the table are weighted for complex survey samples.
